# Values characteristics of Chinese college students with upper-level learning engagement

**DOI:** 10.3389/fpsyg.2025.1414065

**Published:** 2025-03-25

**Authors:** Songge Tang, Di Gao

**Affiliations:** Center for Ideological and Political Education, Northeast Normal University, Changchun, China

**Keywords:** learning engagement, values, Chinese college students, upper-level, cluster analysis

## Abstract

**Introduction:**

Currently, much of the research on learning engagement has more underlined the individual factors associated with levels of learning engagement among college students, but the connections between individual values and learning engagement has not been definitively elucidated. The aim of this research is to reveal the relationships between individual values preferences and degrees of learning engagement, and mainly focuses on the individual values preferences of Chinese college students with upper-level learning engagement.

**Methods:**

Data from 360 first-year Chinese college students majored in foreign languages in Northeast China supported a two-cluster of students based on different learning engagement levels. The assignment of items in the UWES-S scale and the PVQ-21 scale was confirmed through a principal component method to identify the underlying dimensions of Chinese college students’ learning engagement and values. A cluster analysis with K-means algorithm to cluster the participants based on their learning engagement levels. And a series of One-Way Analyses of Variance (ANOVAs) were performed to assess the differences between the cluster groups in relation to each of the values and mainly analyzed individual values characteristics of students with upper-level learning engagement.

**Results:**

Comparing values preferences of students with upper-level learning engagement and those with lower-level learning engagement, the results showed that students with upper-level learning engagement assigned more importance to “Social Focus” values, “Openness to Change” values, benevolence, and hedonism. Which presents a promising opportunity for future research to explore the potential impact of values education on students’ learning engagement.

**Conclusion:**

This research enhances the comprehension of the intricate relationship between learning engagement and values and offers a potential avenue for further investigation into the potential impact of values education on students’ learning engagement.

## 1 Introduction

Students’ learning engagement is an essential component of teaching activities, which has been regarded as the fundamental framework of quality education. In general, learning engagement is defined as a positive, satisfying, and persistent state of learning that is, in which learners can devote themselves to a deep involvement in learning tasks through a collection of mindfully goal-directed behaviors and reflections (Schaufeli et al., 2002; Yin et al., 2022; Ke et al., 2016).

During the past decades, research has highlighted the significance of learning engagement, and demonstrated that learning engagement is an important variable associated with students’ learning performance, academic achievement, individual growth and future development (Finn and Zimmer, 2012; Hsieh, 2014; Wonglorsaichon et al., 2014; Fisher et al., 2021; Lin, 2020; Pedrero and Manzi, 2020). Upper-level learning engagement played a significant role in increasing learning satisfaction and students’ development (Wefald and Downey, 2009; Wang and Fredricks, 2014), while Low-level learning engagement might lead to academic failure and unhealthy behaviors (Li and Lerner, 2011). In terms of the students with upper-level learning engagement, many studies showed that they exhibited superior interpersonal skills, more academic success, less behavioral problems, and lower dropout rates and so on (Wonglorsaichon et al., 2014; Pedrero and Manzi, 2020).

To explore how to effectively improving students’ learning engagement, previous studies have discussed various factors (external and internal factors) that were related to upper-level learning engagement. Not only students’ family socioeconomic status, social factors (e.g., teachers, peers and parents), the critical role of task characteristics, but also students’ gender, age, self-regulated learning and prior knowledge were related to learning engagement (Zhang, 2018; Dong et al., 2020). It is worth mentioning that nowadays, research has more emphasized the individual factors (e.g., personal learning features, personal physical characteristics and personal psychological characteristics) related to college students’ learning engagement (Yang et al., 2018; Zhang, 2018; Wu and Ma, 2022; Ku et al., 2022; Chai et al., 2023).

Individual values play a significant role in individuals’ learning. Previous studies explored individual influence on individuals’ academic achievement (Jones et al., 1990; Cowan et al., 1997), learning approaches (Gamage et al., 2021), learning ability (Lan and Wang, 2023) and so on. Among those, some scholars increased the understanding on the significance of recognizing values as an area of influence on the learning process, thereby, potentially creating a new pathway for the study of learning motivation and engagement (King and Datu 2017; Ku et al., 2022; Ku et al., 2012).

As mentioned above, there has been increasing interest among researchers in studying individual characteristics of students with upper-level learning engagement. However, there is limited literature that deals specifically and explicitly with individual values from the perspectives of students with upper-level learning engagement. Overall, our study contributes to reveal the relationships between learning engagement levels and individual values preferences and excavate the common characteristics of individual values from students with upper-level learning engagement to provide inspirations and reflections for improving students’ learning engagement.

## 2 Research model and hypothesis

### 2.1 Theoretical background

Currently, there are several theories that shed light on the relationship between the learning engagement and personal values. In terms of the impact of personal values on learning engagement, Self-determination theory and expectancy-value theory elucidated that personal values play an important role in students’ learning engagement from different aspects, that is, students’ value beliefs interact and influence their learning performance, engagement, and learning outcomes. In terms of the impact of learning inputs on personal values, social cognitive theory suggests that learning engagement is not merely a resultant of values, but rather a transformative process with the capacity to redefine individuals’ priorities and beliefs. Although, a substantial corpus of research conducted within the purview of the aforementioned theoretical framework has utilized variable-centered analytical techniques to examine linear associations between various variables (e.g., Liu Y et al., 2023, Xie et al., 2022), these approaches may not be sufficient to understand the heterogeneity of different levels of learning engagement. Person-centered approaches, such as latent profile analysis (LPA), offer advantages in clustering students into distinct clusters and exploring their relations with distinct personal values. In recent years, some researchers have adopted person-centered approaches to find the interconnections between learning engagement and personal values, leading to interesting findings (e.g., Lee et al., 2022; Chen et al., 2024).

### 2.2 Research questions

#### 2.2.1 The 3-factor structure of engagement and levels of learning engagement

Learning engagement has been conceptualized from different theoretical perspectives and measured at different dividing dimension (Maican et al., 2021; Zhong et al., 2022; Dong et al., 2023; Kassab et al., 2023; Wong and Liem, 2022). Some studies viewed learning engagement as a multifaceted construct with three dimensions: behavioral engagement, cognitive engagement and emotional engagement (Fredricks et al., 2004; Dong et al., 2020; Pedrero and Manzi, 2020). A measure of college student course engagement with four factors (skills engagement, emotional engagement, participation/interaction engagement, performance engagement) was adapted to assess students’ English learning engagement at the course level (Lin, 2020).

From the perspective of positive psychology, engagement focuses on individual’s positive qualities such as proactive attitudes, optimism, creativity and commitment. Learning engagement has also been considered as a pervasive mind state. Schaufeli et al. (2006) argued that students who were actively engaged in their learning showed a consistent and positive mindset on learning, and they were inclined to sustain this positive statement over time. Hence, they explored the positive mind state comprising elements of energetic effort and psychological resilience, a sense of learning meaningfulness, and full concentration during academic pursuits. These elements align with the dimensions of vigor, dedication, and absorption, respectively. The 3-factor structure of engagement has demonstrated robust psychometric properties and been examined a pivotal tool in assessing learning engagement as across diverse cultural and occupational contexts. Based on Schaufeli’s research, Chinese scholars considered the influence of traditional Chinese culture on and Chinese students’ learning minds and renamed the 3-factor structure of engagement as motivation, energy and absorption (Li and Huang, 2010). In detail, motivation refers to the individual understands the significance of learning and experiences joy in learning. Energy refers to the individual has is not easily tired of working hard for his or her own learning, and is able to persevere in the face of difficulties. Absorption refers to the individual immerses himself/herself in his/her own learning, and achieves a state of selflessness.

*RQ1*: Is the 3-factor structure of learning engagement applicable to Chinese university students?

#### 2.2.2 Characteristics of students with upper-level learning engagement

In Global Learning Qualifications Framework, upper-level learning engagement is approximately defined from the following five aspects: the knowledge identification, skills and abilities, actively participation, feedback utilization and adjustment on their behaviors and learning needs. Studies have confirmed that students with high learning engagement were more enthusiastic and resilient, fully devoted to learning, and more engaged in activities they were interested in Goldman and Swayze (2012).

Considering the positive relationship between influencing factors and learning engagement, research has indicated that positive learning engagement may motivate individuals to actively pursue educational opportunities and access resources, while also cultivating increased determination and diligence, which increased students’ motivation to study (Stephenson and Isaacs, 2019). Xie et al. (2022) contended that there is a strong likelihood of a more significant correlation between deep learning engagement and authentic motivational orientations. Those students more engaged in learning seemed to be motivated and self-regulated learners (Reeve, 2012; Schunk and DiBenedetto, 2022), be more effectiveness, higher intrinsic goals, and less anxiety in the course (Han et al., 2022), and with higher levels of growth mindset (Zhao et al., 2021). Leon et al. (2015) proposed that students who were more actively involved in their learning tended to employ more profound cognitive strategies in their learning process.

*RQ2*: Can Chinese college students can be clustered into different clusters according to the different learning engagement levels?

#### 2.2.3 Influencing factors of learning engagement

Learning engagement is influenced by external and internal factors. In terms of external factors, previous studies have explored a variety of relationships which can influence learning engagement, such as teacher’s support (Liu Q et al., 2023), family socio-economic status (Qiu and Ye, 2023), family capital (Wang and Huang, 2021), the parent–child relationship (Newman et al., 1992), peer relationships (Shao and Kang, 2022), social capital (Dong et al., 2023), and internship environment (Cai et al., 2023).

As for internal factors, previous studies explored the relationships between influencing factors and learning engagement as follows. Some studies found that learning engagement was strongly influenced by prior knowledge (Yang et al., 2018; Dong et al., 2020) and their findings indicate that the relationship between prior knowledge and learner engagement via help-seeking behaviors was heavily influenced by cognitive load (Dong et al., 2020). Some studies found that growth mindset was positively linked with students’ learning engagement (Smith, 2008; Lin-Siegler et al., 2016; Schmidt et al., 2017; Zhao et al., 2021). Chai et al. (2023) found that proactive personality had significantly positive influence on almost all aspects of online learning engagement. Research had also proved that self-regulation had a positive relationship with learning engagement (Stan et al., 2022).

#### 2.2.4 The interconnections between learning engagement and personal values

Values are the fundamental beliefs, behaviors and attitudes that that society has long endorsed and accepted as being right (Gamage et al., 2021). Individual values are the values to which an individual is dedicated and that impact their conduct (Gamage et al., 2021). Values play a significant role in shaping learning engagement (Verplanken and Holland, 2002). The interconnections between personal values and learning engagement reflect how specific value orientations align with distinct aspects of engagement or the students’ degree of learning engagement. The interconnections between personal values and learning engagement can be explained form different theoretical frameworks.

From the perspective of the self-determination theory (SDT), personal values influence learning engagement by satisfying individual’s psychology needs (autonomy, competence, relatedness; Compare et al., 2024). For example, Liu et al. (2023) proposed that there is a positive association between self-concept clarity and learning engagement, emphasizing the significance of fostering students’ self-identity to enhance their engagement in learning activities. Sense of life meaning, an individual’s perspective on the purpose and worth of their existence, has been shown to positively correlate with engagement in learning (Liu et al., 2023). Furthermore, it may indirectly influence learning engagement by shaping an individual’s achievement goal orientation (Xie et al., 2022). When individual’s psychology needs are satisfied, they undergo intrinsic motivation, which is driven by personal interests and values, or experience internalized extrinsic motivation, which aligns with their personal goals. Conversely, when these needs are unmet, motivation and engagement diminish.

From the perspective of expectancy-value theory (EVT), students’ expectancy and value beliefs interact and exerts a significant influence on their academic performance, persistence, and in studies, and subsequent intentions concerning their educational choices (Eccles et al., 1983). Some scholars have clarified the connections the influence of personal values on learning engagement and performance based on this theory. For example, Zhang and Li (2022) argued the relationship between traditional social and cultural values in contemporary China and the “Lying Flat” movement, including students’ low engagement in learning. Fong et al. (2021) conducted an evaluation of expectancy and value beliefs held by United States high school students toward mathematics and science, resulting in the identification of five distinct motivational profiles: low math/low science, moderate math/moderate science, high math/high science, low math/high science, and high math/low science. Their findings revealed that students possessing profiles characterized by higher expectancy and value beliefs attained superior academic performance, as measured by GPA, in both math and science. Notably, the profile exhibiting high expectancy and value beliefs in both subjects demonstrated the highest level of academic persistence.

From the perspective of social cognition theory, the learning environment has a significant impact on learning processes and outcomes, influencing students’ learning behaviors and performances. Students’ personal values may be modulated by teachers, peers, curricula, and the other external factors. For example, digital educational games, providing a contextualized learning environment, improve students’ sense of achievement, satisfaction, and overall enjoyment of the learning process, resulting in a strong engagement of learning (Fokides, 2018). Also, teacher plays a crucial role in motivating career-ready students by offering resources, support, and feedback, helping them acquire learning skills, confidence, and a sense of belonging. Additionally, educators occupy a critical juncture in motivating students to engage in learning through the provision of indispensable resources, supportive guidance, and informative feedback. This facilitation is indispensable in empowering students to acquire learning competencies, enhance their self-confidence, and cultivate a profound sense of belonging (Li et al., 2024). Also, learning engagement promotes self-efficacy to some extent, for mastery experiences in engaging tasks building individual’s confidence, reinforcing values like achievement or self-direction (She et al., 2021; El-Sayad et al., 2021; Cai et al., 2023; Wang et al., 2024).

#### 2.2.5 Values types and learning engagement

Some scholars explored the relationship between values types and learning engagement. For example, Ku et al. (2022) investigated whether higher-order life values (which can also be concluded as social values) were predictors of engagement in the educational process. The researchers conducted a longitudinal study, involving 345 university students of Chinese ethnicity. The study revealed a positive association between higher-order intrinsic life values such as relatedness, self-acceptance, and community, and engagement with learning, above and beyond the effects of materialism. Under the Schwartz’s theoretical framework, research has proven that learning engagement is related to the 10 broad values (hedonism, stimulation, self-direction, security, universalism, benevolence, conformity, tradition, power, and achievement). For example, Çelik and Baturay (2024) argued that improve individual’s learning engagement might promote his/her achievement. Putranti et al. (2024) argued that individual’s engagement was related to social value, benevolence (such as a sustainable, friendly company and collaboration). Zhou et al. (2020) came to a conclusion that reciprocal filial piety (which can be classified as a kind of benevolence) has been proved to be conductive to better academic engagement.

*RQ3*: Is there a significant difference in the value preference of Chinese college students in the Upper-Level Learning Engagement cluster and those in the Lower-Level of Learning Engagement cluster values preferences?

*RQ4*: Which values do Chinese college students with upper-level learning engagement more prefer?

### 2.3 Research model

**Figure 1:**
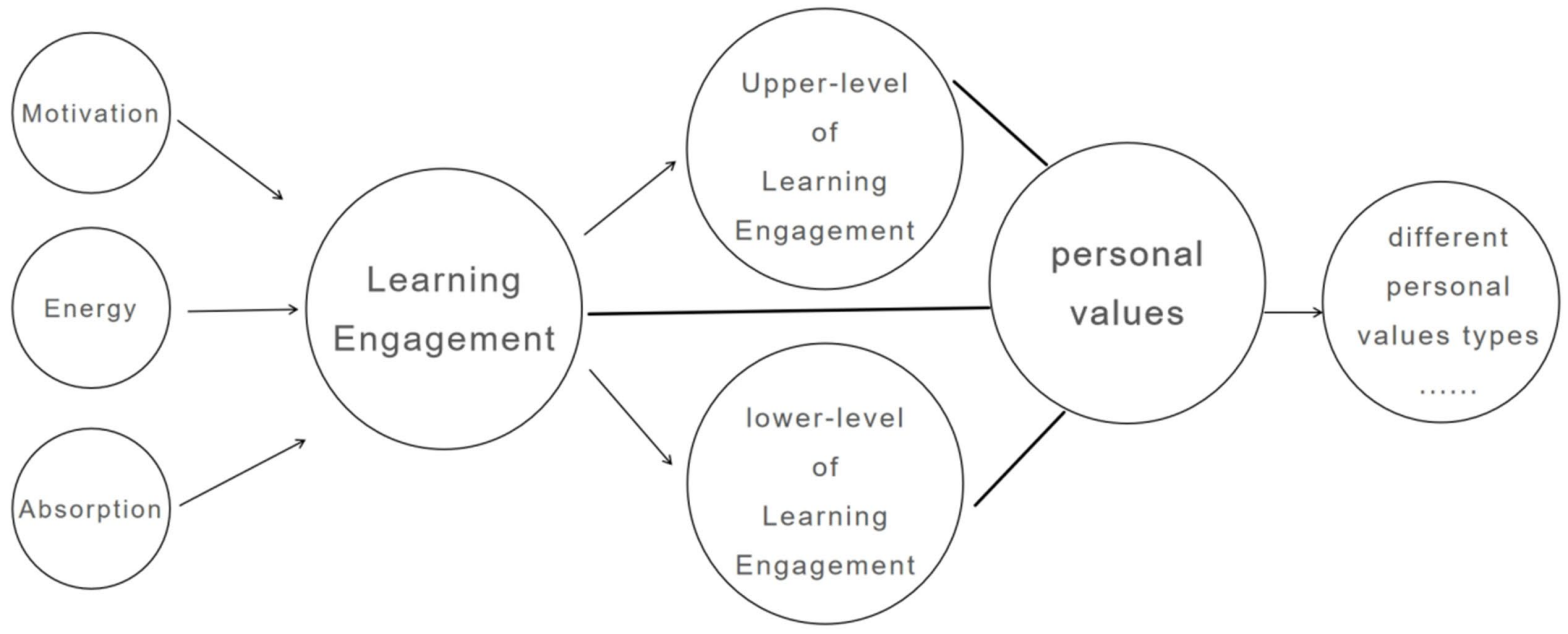
Research model.

The relationship between the two has been explained in the preceding paragraphs as having a certain theoretical basis. Under the framework of 3-factor structure of learning engagement and Schwartz’s values theory, this study adopted latent profile analysis (LPA), and constructed a model to the association among two clusters of different levels of learning engagement and three types of personal values, which can be seen in Figure 1.

## 3 Materials and methods

### 3.1 Participants

This research involved the selection of 360 first-year college students majoring in foreign languages at a private higher education institution in Northeast China, using whole-group convenience sampling for the survey. Researchers and the student instructor cooperated in issuing questionnaires to the participants in classroom settings. Prior to completing the questionnaires, all participants would receive notification regarding the confidentiality of the questionnaires and the research survey’s exclusive use for academic purposes. No rewards or inducements were provided during the process of gathering data. Participants of this study aged from 18 to 22 years old upon surveying (M = 19.26; SD = 0.66). Among the responses, 62 (17.2%) were from boys, and 298 (87.8%) were from girls. With regard to the educational attainment of fathers and mothers, 77.8 and 80.6% had completed high school or less, 10.6 and 11.4% had specialized degrees, 10.8 and 7.5% had a bachelor’s degree, and 0.8 and 0.6% had a master’s degree or above, respectively. As for their native place, 63.6% were from city or town and 36.4% were from rural.

### 3.2 Measure

#### 3.2.1 Learning engagement

Learning engagement was measured by the Utrecht Work Engagement Scale—Student (UWES-S; Schaufeli et al., 2002). The 17 items in the measure are clustered into three dimensions including vigor (6 items, e.g., “I feel energetic when I study”), dedication (5 items, e.g., “I find learning challenging”), and absorption (6 items, e.g., “I am immersed in learning”). A self-administered assessment designed for students to gage the frequency of their experiences of feelings, beliefs, or actions. The scale employs a Likert scale with a range from 0 (Never) to 6 (Every day) for respondents to indicate their level of engagement (Cadime et al., 2016). It has been widely used to evaluate students’ perception of their own learning engagement (Douglas et al., 2016; Wickramasinghe et al., 2018; Maican et al., 2021).

Fang et al. (2008) adapted the Chinese version of the Utrecht Work Engagement Scale-Student (Schaufeli et al., 2002) to evaluate students’ self-assessment of their learning engagement. The modified items had good reliability and validity in the context of Chinese culture (Shao and Kang, 2022; An et al., 2023). Li and Huang (2010) replaced “work” with “learning” in the UWES-S and then revised a Chinese version of UWES-S to meet Chinese college students’ requirements of psychometrics. Since then, Li and Huang (2010) Chinese version of UWES-S has been widely used to assess Chinese college students’ learning engagement (Bao et al., 2022; Yin et al., 2022; Deng et al., 2023). Our study used Li and Huang (2010) Chinese version of UWES-S. Each item was rated on a 7-point Likert scale and the higher the total score, the higher the level of learning engagement (Yin et al., 2022). The Cronbach’s alpha coefficient for this study was calculated to be 0.959.

#### 3.2.2 Portrait values questionnaire

The Schwartz (1992) theory of basic human values proposed 10 broad values (hedonism, stimulation, self-direction, security, universalism, benevolence, conformity, tradition, power, and achievement) and four higher-order value dimensions with different motivational goals. According to the theory, values exist on a continuum at a more fundamental level, and the opposition between different value types can be classified by considering how values are organized into two bi-polar contrasting higher-order dimensions. The first higher-order dimension contrasts self-enhancement and self-transcendence value types: power and achievement values (emphasizing pursuit of self-interests and dominance of others) vs. universalism and benevolence values (underlining concern for the welfare of others). The second higher-order dimension contrasts openness to change and conservation dimensions: self-direction, hedonism and stimulation values (emphasizing independent action, thought and feeling and readiness for new experience) vs. security, conformity and tradition values (underlining self-restriction, order and resistance to change; Schwartz, 2012).

Schwartz created two tools for assessing the 10 fundamental values, with one instrument being more abstract (Schwartz Values Survey, SVS) and the other being less abstract (Portrait Values Questionnaire, PVQ). The Portrait Values Questionnaire (PVQ) inquires about the resemblance to an individual with goals and aspirations (values) as opposed to the resemblance to an individual with specific characteristics. Each portrait delineated an individual’s goals, aspirations, or desires, which tacitly underscore the significance of a particular value. (Schwartz and Rubel, 2005). Davidov (2008) evaluated the appropriateness of the 21-item Portrait Values Questionnaire, which was derived from the original 40-item Portrait Values Questionnaire (PVQ) developed by Schwartz and Rubel (2005) to assess values in the second phase of the European Social Survey (ESS). Gao et al. (2016) revised the 21-item PVQ in Chinese version and analyzed its reliability and validity. Our study used the Gao et al.’s Chinese version of 21-item PVQ and the participants were asked to express their level of agreement with each statement using a rating scale ranging from 1 (strongly agree) to 5 (strongly disagree). The Cronbach’s alpha coefficient in the current study was calculated to be 0.898.

### 3.3 Analysis

Statistical analysis for the study was conducted using SPSS 25.0. First, this study separately analyzes Chinese college students’ learning engagement characteristics and values characteristics. We separately confirmed the assignment of items in the UWES-S scale and the PVQ-21 scale through a principal component method to identify the underlying dimensions of Chinese college students’ learning engagement and values. Then, we applied a cluster analysis with K-means algorithm to cluster the participants based on their learning engagement levels. Next, it further illustrates the common individual values characteristics of students with different learning engagement levels. We examined Chinese college students’ values on a cluster basis and explored the potential relationship between students’ learning engagement and their values characteristics. Ultimately, it compares the values characteristics of students with different learning engagement levels and highlight the common individual values characteristics of those with upper-level learning engagement. A series of One-Way Analyses of Variance (ANOVAs) were performed to assess the differences between the cluster groups in relation to each of the values and mainly analyzed individual values characteristics of students with upper-level learning engagement, so as to infer what kind of value orientation the students with upper-level learning engagement have.

## 4 Results

### 4.1 EFA of the Utrecht work engagement scale-student

The UWES-S is one of the most commonly used instruments to assess students’ engagement in their academic environment. Many studies examine the measurement invariance of the Utrecht Work Engagement Scale-Students (UWES-S) across different cultures through the confirmatory factor analysis (CFA) to support the original three-factor structure (vigor, dedication and absorption) of the UWES-S. For example, employed exploratory factor analysis (EFA) to assess the construct validity of the UWES-S, drawing on data obtained from a cohort of 194 high school seniors in the Kurunegala region of Sri Lanka. In their study, Principal component analysis with Oblimin rotation was utilized in the exploratory factor analysis to establish a three-factor model, which encompassed vigor, dedication, and absorption subscales. This model accounted for 65.4% of the total variance observed in the 16-item UWES-S (with item 13 “When studying, I am very resilient, mentally” deleted). Cadime et al. (2016) used the Portuguese version utilized in the international comparative analysis of Schaufeli et al. (2002) and tested the original three-factor structure (vigor, dedication and absorption) of the UWES-S fit in samples of secondary school pupils (*n* = 251) and university students (*n* = 229). In their study, confirmatory factor analysis (CFA) using Mplus (version 6.1) was employed to assess the consistency of the structure of the UWES-S across both samples, with all items loading on the anticipated dimension in both samples.

Exploratory factor analysis (EFA) would enable researchers to more accurately evaluate the concept validity and measurement invariance of the cross-culturally modified UWES-S. For example, Li and Huang (2010) surveyed about 300 Chinese college students and performed EFA with Oblimin rotation in SPSS 14.0 for Windows, explaining 59.15%, of the total variance for the 17-item UWES-S identifying the three-factor structure of the Chinese version of UWES-S. In their study, they argued that learning in the traditional Chinese culture is different from learning in the western culture. Western students were more inclined to learn spontaneously, out of interest, while Chinese students learned to a certain extent by social and cultural pressures. Hence, Li and Huang (2010) named the three subscales as motivation, energy and absorption according to Chinese college students’ learning characteristic.

In our study, EFA with a principal component method was used to examine the assignment of items on the UWES-S scale and identified the underlying subscales of Chinese college students’ learning engagement. Exploratory factor analyses for all variables were conducted using SPSS (Version 22.0). The standardized deviations for the 17-item UWES-S scale were all within ±2, suggesting the absence of heteroscedasticity.

Based on the three-model on UWES-S (Schaufeli et al., 2002; Li and Huang, 2010), three factors were explicitly extracted using the method of the fixed number of factors. The factor structure of learning engagement was examined and Table 1 reported the solution with varimax (orthogonal) rotation. The final 17-item solution (*α* = 0.959) explained over 70% of the total variance (KMO =0.954; Bartlett’s tests *p* < 0.001), which was appropriate for an exploratory study. As we expected, we found an acceptable fit for the original three-factor structure (motivation, energy and absorption) of the Chinese version of the UWES-S on a sample of Chinese college students.

**Table 1:** Factor structure of the 17-Item categories of the Utrecht work engagement scale-student.

Learning engagement	Communalities	Factor loading (Rotated)		
		Factor 1	Factor 2	Factor 3
I find my studies challenging.	0.706	**0.723**		
My studies inspire me.	0.755	**0.820**		
I am enthusiastic about my studies.	0.804	**0.866**		
I am proud of my studies.	0.647	**0.747**		
I find my studies to be full of meaning and purpose.	0.743	**0.829**		
When I get up in the morning, I feel like going to class.	0.664		**0.402**	
When I study, I feel like I am bursting with energy.	0.760		**0.246**	
Even if the study is not successful, I can persevere.	0.683		**0.251**	
I can continue for a very long time when I am studying.	0.719		**0.415**	
When I’m studying, I feel mentally strong.	0.724		**0.367**	
When studying I feel strong and vigorous.	0.778		**0.220**	
When I am studying, I forget everything else around me.	0.722			**0.426**
Time flies when I’m studying.	0.694			**0.159**
When I study, I only think about studying.	0.760			**0.212**
It’s hard for me to let go of my study.	0.683			**0.277**
I can get carried away by my studies.	0.788			**0.244**
I feel happy when I am studying intensively.	0.685			**0.098**
Percent of common variance explained		25.229%	24.906%	22.306%
Total common variance explained		72.440%		

*N = 360. Extraction Method: Principal Component Analysis. Rotation Method: Varimax with Kaiser Normalization; Rotation converged in eight iterations. Bold values indicates significant dependence of items on relevant factor.*

Through the exploratory factor analysis, our results were generally consistent with previous studies and we named the three subscales as Li and Huang (2010) did. In our result, **the Motivation factor** (α = 0.911) contains five items: *“I find my studies challenging.” “My studies inspire me.” “I am enthusiastic about my studies.” “I am proud of my studies.” “I find my studies to be full of meaning and purpose.”* **The Energy factor** (α = 0.909) contains six items: *“When I get up in the morning, I feel like going to class.” “When I study, I feel like I am bursting with energy.” “Even if the study is not successful, I can persevere.” “I can continue for a very long time when I am studying.” “When I’m studying, I feel mentally strong.” “When studying I feel strong and vigorous.”* **The Absorption factor** (α = 0.913) contains six items: *“When I am studying, I forget everything else around me.” “Time flies when I’m studying.” “When I study, I only think about studying.” “It’s hard for me to let go of my study.” “I can get carried away by my studies.” “I feel happy when I am studying intensively.”*

### 4.2 EFA of the portrait values questionnaire

The Portrait Values Questionnaire has been studied extensively worldwide. Measurement invariance has been observed in several research using the Portrait Values Questionnaire (PVQ-21) to assess fundamental human values across countries and cultures (Davidov, 2008). Davidov (2008) evaluated the appropriateness of PVQ-21 in capturing values through the application of multi-group confirmatory factor analysis (MGCFA) on data obtained from the second round of the European Social Survey (ESS). The study examined the consistency of the values across 25 countries by assessing configural, metric, and scalar invariance.

Several studies also investigated Schwartz’ value orientations in East Asian nations, including China. Heim et al. (2017) conducted a study to analyze the individual value orientations of a sample of Chinese students (*n* = 9,601) using the Schwartz value theory, and compared these findings with those of students from Germany (*n* = 1,118) and Russia (n = 3,890). The researchers performed single group confirmatory factor analyses (CFAs) for each sample and conducted Multi-Group CFA (MGCFA) simultaneously for the three cultural groups and found that in each sample, the four higher-order factors produced satisfactory model fits. Choi et al. (2016) applied principal component analysis (PCA) to Chinese samples based on the Schwartz values. Results of their study revealed five dimensions of individual values among Chinese samples: self-enhancement, universalism, assurance, self-direction, and benevolence.

In our study, we conducted an exploratory factor analysis (EFA) using the principal component factor. The extracted factors were subjected to orthogonal rotation through varimax rotation in order to discern the fundamental dimensions of the participants’ values. For the 21-item of PVQ, the standardized data processing revealed that the standard deviations for the respective items were all within the range of ±2, indicating the absence of heteroscedasticity. According to Kaiser criterion or eigenvalue-greater-than-one rule, four factors’ eigenvalues of exceeding Kaiser’s criterion of 1 and collectively accounted for 56.869% of the variance. However, the fourth factor was discarded because it only included one item related to religion (*“Tradition is important to him. He tries to follow the customs handed down by his religion or his family”*), which was not significant in Chinese social context as the majority of Chinese samples were secular. Finally, a 21-item (*α* = 0.898) three-component structure was retained, explaining over 50% of the total variance (KMO = 0.903; Bartlett’s Tests *p* < 0.001) (Table 2).

Based on the understanding of Schwartz’s 10 fundamental values, we named the 3 components as “Social Focus” (Factor 1; α = 0.866), “Openness to Change” (α = 0.813), and “Self-Enhancement” (α = 0.724).

The “Social Focus” values component encompassing five personal values: security, conformity, tradition, benevolence and universalism, which is largely consistent with Schwartz’s original classification on social focus values. In Schwartz’s theory (Schwartz, 2012), social focus is “concern with outcomes for others or for established institutions” and the conceptual definition components of items in “Social Focus” mainly refers to societal security, compliance with social norms, maintaining cultural and religious traditions, caring for ingroup members and societal concern.

The “Openness to Change” values component containing three individual values: self-direction, hedonism and stimulation, which were totally consistent with those in Schwartz’s original classification. Schwartz (2012) emphasized “Openness to Change” refers to readiness for new ideas, actions, and experiences. Our study similarly follows this interpretation.

“Self-Enhancement” values emphasize pursuing one’s own interests (Schwartz, 2012). Same as the previous one, the items in component “Self-Enhancement” in our results were totally consistent with those in Schwartz’s original classification and the component “Self-Enhancement” encompassed two individual values: power and achievement (Table 2).

**Table 2:** Factor structure of the 21-item categories of the portrait values questionnaire.

Values	Communalities	Factor loading (Rotated)			
		Factor 1	Factor 2	Factor 3	Factor 4
He thinks it is important that every person in the world be treated equally. He believes everyone should have equal opportunities in life.	0.532	**0.607**			
It is important to him to live in secure surroundings. He avoids anything that might endanger his safety.	0.604	**0.493**			
He believes that people should do what they are told. He thinks people should follow rules at all times, even when no one is watching.	0.450	**0.553**			
It is important to him to listen to people who are different from him. Even when he disagrees with them, he still wants to understand them.	0.551	**0.706**			
It is important to him to be humble and modest. He tries not to draw attention to himself.	0.485	**0.694**			
It’s very important to him to help the people around him. He wants to care for their well-being.	0.613	**0.489**			
It is important to him that the government insures his safety against all threats. He wants the state to be strong so it can defend its citizens.	0.436	**0.463**			
It is important to him always to behave properly. He wants to avoid doing anything people would say is wrong.	0.586	**0.603**			
It is important to him to be loyal to his friends. He wants to devote himself to people close to him.	0.630	**0.668**			
He strongly believes that people should care for nature. Looking after the environment is important to him.	0.615	**0.669**			
Thinking up new ideas and being creative is important to him. He likes to do things in his own original way.	0.588		**0.684**		
He likes surprises and is always looking for new things to do. He thinks it is important to do lots of different things in life.	0.593		**0.738**		
Having a good time is important to him. He likes to ‘spoil’ himself.	0.571		**0.525**		
It is important to him to make his own decisions about what he does. He likes to be free to plan and not depend on others.	0.418		**0.561**		
He looks for adventures and likes to take risks. He wants to have an exciting life.	0.635		**0.747**		
He seeks every chance he can to have fun. It is important to him to do things that give him pleasure.	0.611		**0.682**		
It is important to him to be rich. He wants to have a lot of money and expensive things.	0.587			**0.748**	
It’s important to him to show his abilities. He wants people to admire what he does.	0.544			**0.641**	
Being very successful is important to him. He hopes people will recognize his achievements.	0.618			**0.609**	
It is important to him to get respect from others. He wants people to do what he says.	0.685			**0.591**	
Tradition is important to him. He tries to follow the customs handed down by his religion or his family.	0.591				**0.756**
Percent of common variance explained		19.231%	17.858%	12.785%	6.996%
Total common variance explained		56.869%			

*N = 360. Extraction Method: Principal Component Analysis. Rotation Method: Varimax with Kaiser Normalization; Rotation converged in six iterations. Bold values indicates significant dependence of items on relevant factor.*

### 4.3 Chinese college students’ clusters on learning engagement

To reiterate, our second research question asked whether college students could be meaningfully clustered by their different learning engagement levels. To achieve this objective, a K-means cluster analysis was applied for this purpose to students’ scores on the learning engagement components derived from an EFA. To find a fixed number (k) of clusters in a dataset, we ran solutions with K = 2, 3, 4, 5, 6, 7, 8 and 9, respectively, and compared these solutions by analyzing *p* values from ANOVA, multiple comparison tests and iteration tables. After going through the above operations, a 2-cluster solution was identified [Motivation *F* (2, 357) = 722.738, *p* = 0.000; Energy F (2, 357) = 0.588, *p* = 0.444; Absorption *F* (2, 357) = 1.946, *p* = 0.164]. Table 3 shows the ANOVA results on the three components of learning engagement ratings by cluster.

**Table 3:** ANOVA on the three components of learning engagement by cluster.

		Sum of squares	df	Mean Square	F	Sig.
Motivation	Between Groups	240.079	1	240.079	722.738	0.000
	Within Groups	118.921	358	0.332		
	Total	359.000	359			
Energy	Between Groups	0.589	1	0.589	0.588	0.444
	Within Groups	358.411	358	1.001		
	Total	359.000	359			
Absorption	Between Groups	1.941	1	1.941	1.946	0.164
	Within Groups	357.059	358	0.997		
	Total	359.000	359			

Table 4 lists means of Chinese college students’ ratings on different aspects of learning engagement by cluster. The means on all components (Motivation component, Energy component and Absorption component) of learning engagement in Cluster 2 were obviously higher than those of Cluster 1. Thus, we labeled Cluster 1 as Lower-Level of Learning Engagement cluster (LLLE), Cluster 2 as Upper-Level Learning Engagement cluster (ULLE).

**Table 4:** Means of Chinese college students’ learning engagement by cluster.

Component	Cluster 1 (*n* = 169)	Cluster 2 (*n* = 191)
Motivation	4.11	5.59
Energy	3.95	4.72
Absorption	4.25	5.11

#### 4.3.1 Lower-level of learning engagement cluster (LLLE, *n* = 169)

We labeled Cluster 1 as lower-level of learning engagement cluster. The number of participants in this cluster is above half of the total number. Mean values on the three dimensions of learning engagement of Cluster 1 are all lower than those of Cluster 2. This shows that participants in this cluster have the lower level of learning engagement and low engage in all aspects of learning. Therefore, this cluster is determined as a lower-level of learning engagement cluster.

#### 4.3.2 Upper-level learning engagement cluster (ULLE, *n* = 191)

Based on the mean values exhibited in Table 4, we labeled Cluster 2 as upper-level of learning engagement cluster. The number of participants in this cluster is over half of the total number and is a little higher than that of Cluster 1. Comparing with the Cluster 1, mean ratings of this cluster on the three components of learning engagement are all higher. The above indicates that participants in this cluster have a higher degree of learning engagement and highly engage in all aspects of learning. Hence, this cluster is determined as an upper-level of learning engagement cluster.

### 4.4 Differences in values by cluster

To explore whether Chinese college students with different learning engagement levels differ in their individual values, we separately calculated the means of “Social Focus” values, “Openness to Change” values, and “Self-Enhancement” values on a cluster base. Table 5 lists means of the above three groups of values by cluster.

**Table 5:** Means on the three types of values by cluster.

Component		Lower-level of learning engagement (Cluster 1, *n* = 169)	Upper-level learning engagement (Cluster 2, *n* = 191)
Social focus values	Security	3.53	3.86
	Conformity	3.36	3.70
	Tradition	3.41	3.64
	Benevolence	3.52	4.08
	Universalism	3.47	3.95
	Total	**3.47**	**3.88**
Openness to change values	Self-direction	3.31	3.81
	Hedonism	3.59	4.11
	Stimulation	3.31	3.69
	Total	**3.41**	**3.87**
Self-enhancement values	Power	3.24	3.35
	Achievement	3.37	3.68
	Total	**3.31**	**3.52**

Bold values indicates the total values on each cluster of the specific component.

A series of One-Way Analyses of Variance (ANOVAs) were then applied to compare the means of three types of values among three clusters, respectively. Table 6 shows the descriptives on the three values ratings by cluster. Table 7 shows the ANOVA results on the three values ratings by cluster.

**Table 6:** Descriptives on the three values ratings by cluster.

Dependent variable	Cluster	N	Mean	Std. deviation	Std. error	95% Confidence Interval	
						Lower bound	Upper bound
Social focus values	LLLE	169	−0.3215349	0.93261323	0.07173948	−0.4631619	−0.1799079
			0.2844994	0.97318666	0.07041732	0.1455993	0.4233996
	ULLE	191	0.0000000	1.00000000	0.05270463	−0.1036486	0.1036486
			−0.3507074	0.87869101	0.06759162	−0.4841457	−0.2172690
	Total	360	0.3103117	1.00028747	0.07237826	0.1675436	0.4530799
			0.0000000	1.00000000	0.05270463	−0.1036486	0.1036486
Openness to change values	LLLE	169	−0.0213401	0.88539798	0.06810754	−0.1557970	0.1131168
			0.0188821	1.09341274	0.07911657	−0.1371776	0.1749417
	ULLE	191	0.0000000	1.00000000	0.05270463	−0.1036486	0.1036486
			−0.3215349	0.93261323	0.07173948	−0.4631619	−0.1799079
	Total	360	0.2844994	0.97318666	0.07041732	0.1455993	0.4233996
			0.0000000	1.00000000	0.05270463	−0.1036486	0.1036486
Self-enhancement values	LLLE	169	−0.3507074	0.87869101	0.06759162	−0.4841457	−0.2172690
			0.3103117	1.00028747	0.07237826	0.1675436	0.4530799
	ULLE	191	0.0000000	1.00000000	0.05270463	−0.1036486	0.1036486
			−0.0213401	0.88539798	0.06810754	−0.1557970	0.1131168
	Total	360	0.0188821	1.09341274	0.07911657	−0.1371776	0.1749417
			0.0000000	1.00000000	0.05270463	−0.1036486	0.1036486

**Table 7:** ANOVA on the three values ratings by cluster.

ANOVA						
		Sum of squares	df	Mean square	F	Sig.
Social focus values	Between Groups	32.932	1	32.932	36.156	0.000
	Within Groups	326.068	358	0.911		
	Total	359.000	359			
Openness to change values	Between Groups	39.178	1	39.178	43.855	0.000
	Within Groups	319.822	358	0.893		
	Total	359.000	359			
Self-enhancement values	Between Groups	0.145	1	0.145	0.145	0.704
	Within Groups	358.855	358	1.002		
	Total	359.000	359			

Table 7 demonstrates that there are significant differences on all examined 3 types of values between two clusters. By looking at means and ANOVA results, we analyzed the inter-cluster differences by values, respectively. As for “Social Focus” values, there are significant differences on students’ ratings among three clusters [*F* (2, 357) = 36.156, *p* = 0.000]. Table 5 shows that on “Social Focus” values, Upper-Level Learning Engagement cluster (ULLE) have higher means than Lower-Level of Learning Engagement cluster (LLLE; ULLE, M = 3.88, SD = 0.85; LLLE, M = 3.47, SD = 0.75, *p* = 0.000). This indicates that Chinese college students with upper-level of learning engagement engage in all dimensions of learning engagement and attach greater importance to the “Social Focus” values than those with lower-levels of learning engagement. Considering five individual values of “Social Focus” values (that is, Security, Conformity, Tradition, Benevolence, Universalism), means on them of students in ULLE cluster were all higher than those of students in LLLE cluster. It should be noted that mean value of Benevolence in ULLE is obviously high (M > 4), indicating that students with upper-level of learning engagement assigned greater importance on caring for ingroup members.

In terms of “Openness to Change” values, we found that there were also significant differences between two clusters [F (2, 357) = 43.855, *p* = 0.000] as shown in Tables 6, 7. By examining their mean values and the results from comparison, we found that students in ULLE cluster attached higher importance on “Openness to Change” values than those in LLLE cluster (ULLE, M = 3.87, SD = 0.88; LLLE, M = 3.41, SD = 0.80 *p* = 0.000). Considering three individual values of “Openness to Change” values, means on Self-direction, Hedonism and Stimulation of students in ULLE cluster were all higher than those in LLLE cluster. Also, it should be noted that mean value of Hedonism in ULLE is obviously high (M > 4), indicating that students with upper-level of learning engagement assigned more importance on pleasure and sensuous gratification for themselves.

As for “Self-Enhancement” values, the differences between two clusters [F (2, 357) = 0.145, *p* = 0.704] were not so significant. However, similar to the above two types of values, students in ULLE cluster reported higher ratings than students in LLLE cluster (ULLE, M = 3.31. SD = 0.95; LLLE, M = 3.52, SD = 0.78, *p* = 0.704). Considering two individual values of “Self-Enhancement” values, means on Power and Achievement of students in ULLE cluster were just a little higher than those in LLLE cluster.

The above data and analysis proved that students with different learning engagement levels attached different degrees of importance to “Social Focus” values, “Openness to Change” values and “Self-Enhancement” values. Our results came to a conclusion that Chinese college students with upper-level of learning engagement attached more importance to these three values, especially for “Social Focus” values and “Openness to Change” values. Also, it is worth noting that for concrete individual values, Chinese college students with upper-level of learning engagement highly value benevolence and hedonism (respectively rating 4.08 and 4.01, meaning almost completely agree to this type of value).

## 5 Discussion

As discussed in the preceding paragraphs, upper-level of learning engagement contributes to students’ learning performance, academic achievement and personal development. A variety of external factors and internal factors has been verified relating to learning engagement. This study sought to examine the relationship between learning engagement and individual values, and aimed to explore students with upper-level of learning engagement more prefer what kind of individual values.

In this paper, we first used exploratory factor analysis (EFA) to analyze the main characteristics of the learning engagement and values of Chinese college students, discovering that Chinese college students’ learning engagement could be characterized by motivation, energy, absorption, and their individual values could be characterized by “Social Focus” values, “Openness to Change” values and “Self-Enhancement” values. For a better understanding on the learning engagement and values of our sample, we further classified these students into two clusters through the cluster analysis with K-means algorithm. The clusters of volunteers were classified by the distinct intensity levels they engage in learning, namely Upper-Level Learning Engagement cluster (ULLE), and Lower-Level of Learning Engagement cluster (LLLE).

### 5.1 Upper-level learning engagement and social focus values

One of the interesting findings of our study is that students with upper-level learning engagement attach more importance to social focus values rather than personal focus values. Consistent with previous studies, our results support values on social focus positively predicted behavioral, emotional, and cognitive academic engagement in school (King et al., 2012). For instance, developing educational programs to increase medical students’ social concern could lead to individual expertise development (Park et al., 2017). Those who have a hard time integrating with other members have lower academic performance, whereas the contrary is true of those who relate successfully (Walters and Bowen, 1997).

To some extent, assigning greater importance on social focus values may lead to the fulfillment of psychological needs of the participants in our study. From the perspective of self-determination theory (SDT), “Human beings can be proactive and engaged or, alternatively, passive and alienated, largely as a function of the social conditions in which they develop and function” (Deci and Ryan, 2000). Intrinsic goals lead to positive effects for satisfying three psychological needs of autonomy, competence, and relatedness. For example, focusing on social concern may satisfy medical students’ the greatest fulfillment of psychological needs (Park et al., 2017). The relationship between upper-level learning engagement and social focus values may be partly accounted by autonomy need satisfaction. Findings indicated that the fulfillment of autonomy can positively predict academic engagement (Peters et al., 2007; Diseth et al., 2012) and argued that the association between values (such as reciprocal filial piety) and students’ academic development can be partially explained by the satisfying of their autonomy needs, which is a universal finding across different cultural backgrounds (King et al., 2012). When someone has a higher degree of autonomy, they typically feel more in control of their own life and destiny and are more likely to actively participate in activities out of real interest and internal values rather than due to outside pressure. Besides, the socially oriented achievement motive (a desire to meet expectations of significant others) may even be more salient in collectivist settings where individuals are prone to construe themselves in a more interdependent manner (Markus and Kitayama, 1991). For example, research on Filipino culture generally suggested that there are strong needs for social acceptance and group belonging (Bulatao, 1962), and social reasons for learning are relatively important for students in the Philippines (Bernardo, 2008).

In China, the Confucian values transmitted in the Chinese culture put much emphasis on harmonious interpersonal relations (Na et al., 2010) and collective identity (Li et al., 2019) as an important basis of social obligations and personal meanings. Confucian ethics, which emphasize harmonious relationships and social stability, is typified by a hierarchical social system focused on the family and ideals based on clans (Jingjit and Fotaki, 2010). In Confucian tradition, instead of being viewed as a separate, rational, and competitive entity from social groups, the individual (self) is seen as relational and plays certain social roles within a network of interactions and only in a community where relationships are regulated can he grow into a decent person (Woods and Lamond, 2011). The individual (self) is inseparable from the group. Given its emphasis on individuals’ obligations and responsibility for the groups, focusing on the results of others or groups may lead to the fulfillment of psychological needs of Chinese students.

### 5.2 Upper-level learning engagement and openness to change values

Parallel to the above finding, we confirmed that students with upper-level learning engagement attach more importance to openness to change values, including self-direction, hedonism, stimulation. This indicates that students more engaged in learning are more inclined to be open to embracing new concepts, behaviors, and opportunities. For example, Van den Broeck et al. (2016) demonstrated that stimulation and self-direction correlated strongly with absorption. Davis et al. (2024) observed that while participants exhibited a high level of motivation to engage, their engagement was hindered when they had low levels of professional autonomy. Furthermore, some participants encountered additional challenges in their engagement due to academic uncertainty. They contended that providing scaffolded strategies for self-direction are crucial in supporting learner engagement.

Openness to change values is related to students’ learning engagement by shaping their satisfaction of autonomy. When educators support students’ autonomy, educators can empower them to engage in a learning process that is driven by their own interests and motivations. Take self-directed learning as an example, students set their own learning goals, monitor their progress, and reflect on their learning experiences. In this way, they can develop a sense of agency and initiative that will serve them well throughout their academic and personal lives.

### 5.3 Upper-level learning engagement and benevolence

The current study uncovers the relationship between upper-level learning engagement and benevolence. Previous studies affirmed the relationship between benevolence and learning. Gázquez et al. (2015) proved that students who showed high benevolence had better academic performance. In Brown (2024)’s dissertation, he argued that benevolence, honesty, openness, reliability, and competency constitutes trust, and believed trust is “trust is an important element in human learning because much of what is learned is based on the verbal and written statements of others that the learner is asked to believe [often] without independent evidence.” Also, he examined the relationship trust and writing played in their writing-intensive classrooms.

The study has successfully examined benevolence is an important factor related to the learning engagement. Also, the learning engagement may be a transformative process to foster students’ benevolence value. The interaction between the two will not be discussed in this study. The focus of this section needs to shed light on why Chinese college students with upper-level of learning engagement more prefer to the benevolence value. Their high prevalence of benevolence is inextricably linked to the influence of traditional Confucian culture. Confucian benevolence contains the connotation of Schwartz’s benevolence. Schwartz’s benevolence is defined as the preservation and strengthening of others’ wellbeing (Schwartz and Bilsky, 1987). In Confucian system, “benevolence” (Ren) is not only one of the most major concepts of virtues, but is considered as the highest moral principle. For example, in the Xue Er of the Analects of Confucius, benevolence is defined as “A youth, when at home, should be filial, and abroad, respectful to his elders. He should overflow in love to all, and cultivate the friendship of the good.” As a moral principle, benevolence regulates how people study, how they behave and how they handle interpersonal relationships.

More broadly, the notion of benevolence is not prominently known nor considered a pedagogical necessity for improving learning engagement. In fact, benevolence exists in teacher-student relationship, peer relationship, teaching courses and learning environment. In anticipation of future developments, there is a promising prospect for the integration of benevolence-promotion initiatives with supplementary support services, including counseling and academic advising, ultimately establishing a resilient and comprehensive framework to enhance student engagement and learning support.

### 5.4 Upper-level learning engagement and hedonism

One of the most remarkable findings that emerged from the study is the existence on the association between hedonism and upper-level learning engagement. Liu (2006) defined hedonism as “the belief that sensual pleasures can make a person happy and satisfied, and that such pleasures are the purpose of life.” This study proves that comparing to students with lower-level of learning engagement, students with upper-level learning engagement assigned the higher importance to hedonism.

Previous researches suggested that hedonism had a negative effect on learning. For instance, Lietz and Matthews (2010) carried out a three-year longitudinal research study on “the impact of values and learning approaches on student achievement: gender and academic discipline influences” using a cohort of international students at a university in Germany. They found that hedonism was negatively related to approach across all occasions and students who valued hedonism (that is, the tendency to have fun and a good time) were less likely to follow the achieving and deep approach (that is, two types of learning approaches). Koscielniak and Bojanowska (2019) investigated 219 Polish university students using Schwartz’s Portrait of Values Questionnaire. They revealed that the relationships of some values with academic dishonesty were significantly moderated by students’ academic performance and hedonism was significantly positively correlated with academic cheating tendencies.

Conversely, as previously described, Chinese college students who demonstrated a higher engagement in learning are identified to a greater extent with the hedonism value. This finding is consistent with the argument that hedonism is a fundamental component in successful learners’ engagement (Tsang, 2023). There is a good rationale explaining such phenomenon from a psychological perspective. College students do not seek out to engage in learning for instrumental purposes; rather, they simply enjoy studying for its own sake. Their pursuit of such pleasure is linked to the state of flow, namely when one is “completely involved in something to the point of forgetting time, fatigue, and everything else but the activity itself” (Csikszentmihalyi and Rathunde, 1993, p. 59).

### 5.5 Contributions

This study makes several contributions in the following aspects. First, this study enriches the literature on exploring college students’ learning engagement characteristics and values characteristics in a Chinese cultural context. Second, this study extends the current understanding of the relations between learning engagement levels and college students’ individual values preference. Additionally, the study uncovers the values preferences of students with upper-level learning engagement, comparing to those with lower-level learning engagement. Third, this study also shed some light into the upper-level learning engagement and several values, such as social focus values, benevolence and hedonism. Finally, a key implication of the study is the recognition that the values preferences of students with upper-level learning engagement and those with lower-level learning engagement are obviously different. It also broadens college teachers and administrators’ understanding of promoting students’ positive learning engagement, performance and outcomes through values education.

### 5.6 Limitations and further directions

Despite the above contributions, this study also has several limitations. First, we recruited first-year college students majored in foreign languages in a private higher learning institution in Northeast China as participants in this study. When generalizing the findings of this study to other groups, caution should be used. It is worth exploring the study of research subjects of different cultural contexts or developmental stages.

Second, the current study demonstrates that the exploratory factor analysis on UWES-S and PVQ-21 scales is a valid method for assessing degrees of learning engagement and values characteristics among Chinese college students. Influenced by the cultural and societal norms of China, students may interpret certain items in the UWES-S and PVQ-21 scales differently, potentially impacting the scores of these items in the Chinese-language versions of the scales. Subsequent studies may delve deeper into the disparities in the interpretation of individual items on the scale between Chinese individuals and those from other countries.

Next, the study focuses on revealing the values preferences of student groups with different degrees of learning engagement and neglects the learning motivations influence on the learning engagement. Values preferences of students with with upper-level learning engagement may be related to their learning motivations. Future research can explore the relationship between students’ learning motivation, learning engagement and values preferences, as well as the learning engagement degree and values characteristics of students with different learning motivations. In terms of interventions to enhance students’ engagement, more targeted recommendations can also be made for students with different motivations. For example, the classification system of motivational behaviors developed by Ahmadi et al. (2023) is useful for increasing learning engagement for students with different motivations. Also, they pointed that discussing class values could improve motivations, which might satisfy individuals’ psychological needs and improve their engagement.

Finally, this study did not discuss the influence of artificial intelligence (AI) on students’ learning engagement. In recent years, new AI tools has significantly facilitated the process of transcending traditional classroom boundaries, thereby fostering more interactive and engaging learning experiences. Many scholars have conducted research indicating that AI, employed as an instructional tool, holds the potential to significantly enhance the learning performance, engagement motivations of students (Huang et al., 2023; AlTwijri and Alghizzi, 2024; Youssef et al., 2024). Ravšelj et al. (2025) found that observed predominantly positive sentiments among students using ChatGPT, with curiosity and calmness emerging as the most common emotions. Further research could discuss if AI can influence the relationship between learning engagement and values preferences, for example, future studies could explore students’ values preferences when they use AI to enhance engagement in learning over time.

## 6 Conclusion

In brief, our study has reveals that Chinese college students exhibit varying degrees of learning engagement levels, which in turn influences their prioritization of “Social Focus” values, “Openness to Change” values, and “Self-Enhancement” values. A significant discovery from our research is that students with higher levels of learning engagement tend to more emphasize “Social Focus” values, “Openness to Change” values, benevolence, and hedonism. This research enhances our comprehension of the intricate relationship between learning engagement and values. Additionally, this study offers a potential avenue for further investigation into the potential impact of values education on students’ learning engagement.

## Data Availability

The raw data supporting the conclusions of this article will be made available by the authors, without undue reservation.
